# Supplementary material for: Heparin-based hydrogel scaffolding alters the transcriptomic profile and increases the chemoresistance of MDA-MB-231 triple-negative breast cancer cells
Source: Biomater Sci. 2020 Feb 13;8(10):2786–96. doi: 10.1039/c9bm01481k (PMC7497406; doi:10.1039/c9bm01481k)
Supplement: Supplementary file 2 [file BM-008-C9BM01481K-s002.zip › Supplementary File 4/EGFvControl/Pathways/my_analysis.Gsea.1545200981068/HALLMARK_IL2_STAT5_SIGNALING.html]

Details for gene set HALLMARK\_IL2\_STAT5\_SIGNALING[GSEA]

|  || Dataset | expr.class.cls#EGF\_versus\_CONTROL.class.cls#EGF\_versus\_CONTROL\_repos |
| Phenotype | class.cls#EGF\_versus\_CONTROL\_repos |
| Upregulated in class | CONTROL |
| GeneSet | HALLMARK\_IL2\_STAT5\_SIGNALING |
| Enrichment Score (ES) | -0.41131428 |
| Normalized Enrichment Score (NES) | -1.9508168 |
| Nominal p-value | 0.0 |
| FDR q-value | 4.914693E-4 |
| FWER p-Value | 0.003 |
Table: GSEA Results Summary

  

Fig 1: Enrichment plot: HALLMARK\_IL2\_STAT5\_SIGNALING      
 Profile of the Running ES Score & Positions of GeneSet Members on the Rank Ordered List

  

| PROBE | DESCRIPTION (from dataset) | GENE SYMBOL | GENE\_TITLE | RANK IN GENE LIST | RANK METRIC SCORE | RUNNING ES | CORE ENRICHMENT || 1 | NOP2 | na |  |  | 166 | 2.175 | 0.0054 | No |
| 2 | UCK2 | na |  |  | 168 | 2.173 | 0.0195 | No |
| 3 | SLC1A5 | na |  |  | 171 | 2.171 | 0.0335 | No |
| 4 | CCNE1 | na |  |  | 408 | 1.841 | 0.0331 | No |
| 5 | ODC1 | na |  |  | 415 | 1.835 | 0.0447 | No |
| 6 | CDC6 | na |  |  | 472 | 1.786 | 0.0534 | No |
| 7 | UMPS | na |  |  | 526 | 1.756 | 0.0621 | No |
| 8 | PNP | na |  |  | 546 | 1.742 | 0.0724 | No |
| 9 | SLC39A8 | na |  |  | 661 | 1.665 | 0.0772 | No |
| 10 | ALCAM | na |  |  | 834 | 1.575 | 0.0785 | No |
| 11 | CASP3 | na |  |  | 1155 | 1.458 | 0.0711 | No |
| 12 | BCL2 | na |  |  | 1163 | 1.454 | 0.0802 | No |
| 13 | LRRC8C | na |  |  | 1164 | 1.453 | 0.0897 | No |
| 14 | PDCD2L | na |  |  | 1192 | 1.444 | 0.0977 | No |
| 15 | GADD45B | na |  |  | 1283 | 1.417 | 0.1022 | No |
| 16 | FAM126B | na |  |  | 1289 | 1.415 | 0.1111 | No |
| 17 | PHTF2 | na |  |  | 1774 | 1.274 | 0.0940 | No |
| 18 | CCND3 | na |  |  | 2241 | 1.169 | 0.0771 | No |
| 19 | HIPK2 | na |  |  | 2290 | 1.159 | 0.0821 | No |
| 20 | PUS1 | na |  |  | 2524 | 1.107 | 0.0771 | No |
| 21 | MAP6 | na |  |  | 2528 | 1.106 | 0.0841 | No |
| 22 | LCLAT1 | na |  |  | 2685 | 1.080 | 0.0830 | No |
| 23 | GUCY1B3 | na |  |  | 2691 | 1.078 | 0.0897 | No |
| 24 | SOCS2 | na |  |  | 2850 | 1.053 | 0.0883 | No |
| 25 | F2RL2 | na |  |  | 3255 | 0.977 | 0.0734 | No |
| 26 | CDC42SE2 | na |  |  | 3509 | 0.935 | 0.0662 | No |
| 27 | PRKCH | na |  |  | 3569 | 0.925 | 0.0691 | No |
| 28 | AHCY | na |  |  | 3594 | 0.921 | 0.0739 | No |
| 29 | MYC | na |  |  | 3907 | 0.866 | 0.0631 | No |
| 30 | CD44 | na |  |  | 3954 | 0.860 | 0.0663 | No |
| 31 | ARL4A | na |  |  | 4169 | 0.826 | 0.0604 | No |
| 32 | TTC39B | na |  |  | 4715 | 0.745 | 0.0367 | No |
| 33 | LRIG1 | na |  |  | 4830 | 0.725 | 0.0354 | No |
| 34 | S100A1 | na |  |  | 4847 | 0.723 | 0.0393 | No |
| 35 | PRNP | na |  |  | 4977 | 0.711 | 0.0371 | No |
| 36 | BATF3 | na |  |  | 5685 | 0.612 | 0.0040 | No |
| 37 | SNX14 | na |  |  | 5687 | 0.611 | 0.0079 | No |
| 38 | DCPS | na |  |  | 5744 | 0.604 | 0.0089 | No |
| 39 | ITGAE | na |  |  | 6279 | 0.530 | -0.0157 | No |
| 40 | POU2F1 | na |  |  | 6356 | 0.520 | -0.0163 | No |
| 41 | FURIN | na |  |  | 6470 | 0.506 | -0.0190 | No |
| 42 | TGM2 | na |  |  | 6481 | 0.505 | -0.0162 | No |
| 43 | GLIPR2 | na |  |  | 7235 | 0.417 | -0.0530 | No |
| 44 | PTRH2 | na |  |  | 7306 | 0.407 | -0.0541 | No |
| 45 | NCS1 | na |  |  | 7418 | 0.394 | -0.0573 | No |
| 46 | MYO1C | na |  |  | 7509 | 0.385 | -0.0595 | No |
| 47 | SWAP70 | na |  |  | 7541 | 0.380 | -0.0587 | No |
| 48 | CD83 | na |  |  | 7756 | 0.356 | -0.0676 | No |
| 49 | AKAP2 | na |  |  | 8129 | 0.314 | -0.0851 | No |
| 50 | PLSCR1 | na |  |  | 8332 | 0.290 | -0.0938 | No |
| 51 | TWSG1 | na |  |  | 8371 | 0.286 | -0.0940 | No |
| 52 | ITGA6 | na |  |  | 8611 | 0.260 | -0.1048 | No |
| 53 | SYT11 | na |  |  | 9368 | 0.177 | -0.1434 | No |
| 54 | GSTO1 | na |  |  | 9438 | 0.165 | -0.1459 | No |
| 55 | GPR83 | na |  |  | 9873 | 0.119 | -0.1679 | No |
| 56 | BMPR2 | na |  |  | 9896 | 0.116 | -0.1683 | No |
| 57 | DENND5A | na |  |  | 10268 | 0.073 | -0.1873 | No |
| 58 | PTCH1 | na |  |  | 10340 | 0.063 | -0.1907 | No |
| 59 | FGL2 | na |  |  | 10347 | 0.062 | -0.1906 | No |
| 60 | ENO3 | na |  |  | 10445 | 0.059 | -0.1953 | No |
| 61 | NT5E | na |  |  | 10569 | 0.043 | -0.2015 | No |
| 62 | EOMES | na |  |  | 10664 | 0.030 | -0.2062 | No |
| 63 | RABGAP1L | na |  |  | 10691 | 0.026 | -0.2074 | No |
| 64 | GALM | na |  |  | 11019 | -0.006 | -0.2245 | No |
| 65 | NCOA3 | na |  |  | 11832 | -0.104 | -0.2665 | No |
| 66 | IKZF2 | na |  |  | 11901 | -0.114 | -0.2693 | No |
| 67 | RHOH | na |  |  | 12101 | -0.136 | -0.2789 | No |
| 68 | BCL2L1 | na |  |  | 12327 | -0.159 | -0.2897 | No |
| 69 | CYFIP1 | na |  |  | 12443 | -0.177 | -0.2946 | No |
| 70 | IRF6 | na |  |  | 12527 | -0.188 | -0.2977 | No |
| 71 | LIF | na |  |  | 12907 | -0.232 | -0.3161 | No |
| 72 | ST3GAL4 | na |  |  | 12928 | -0.236 | -0.3156 | No |
| 73 | MAPKAPK2 | na |  |  | 13030 | -0.248 | -0.3193 | No |
| 74 | PTGER2 | na |  |  | 13046 | -0.250 | -0.3185 | No |
| 75 | RNH1 | na |  |  | 13095 | -0.256 | -0.3193 | No |
| 76 | RRAGD | na |  |  | 13146 | -0.265 | -0.3202 | No |
| 77 | APLP1 | na |  |  | 13196 | -0.273 | -0.3210 | No |
| 78 | RGS16 | na |  |  | 13422 | -0.305 | -0.3308 | No |
| 79 | TNFRSF1B | na |  |  | 13603 | -0.331 | -0.3381 | No |
| 80 | FAH | na |  |  | 13686 | -0.343 | -0.3402 | No |
| 81 | CAPN3 | na |  |  | 13801 | -0.349 | -0.3439 | No |
| 82 | CXCL10 | na |  |  | 13845 | -0.359 | -0.3439 | No |
| 83 | AGER | na |  |  | 13884 | -0.363 | -0.3435 | No |
| 84 | BATF | na |  |  | 13925 | -0.367 | -0.3432 | No |
| 85 | KLF6 | na |  |  | 14086 | -0.390 | -0.3491 | No |
| 86 | CKAP4 | na |  |  | 14148 | -0.399 | -0.3497 | No |
| 87 | HK2 | na |  |  | 14409 | -0.434 | -0.3605 | No |
| 88 | CAPG | na |  |  | 14567 | -0.458 | -0.3658 | No |
| 89 | IL3RA | na |  |  | 15196 | -0.545 | -0.3952 | No |
| 90 | MXD1 | na |  |  | 15373 | -0.577 | -0.4007 | No |
| 91 | SPRED2 | na |  |  | 15392 | -0.581 | -0.3979 | No |
| 92 | IGF2R | na |  |  | 15552 | -0.602 | -0.4023 | No |
| 93 | SH3BGRL2 | na |  |  | 15664 | -0.617 | -0.4041 | No |
| 94 | SYNGR2 | na |  |  | 15687 | -0.624 | -0.4012 | No |
| 95 | PHLDA1 | na |  |  | 15776 | -0.644 | -0.4016 | No |
| 96 | SCN9A | na |  |  | 15835 | -0.654 | -0.4004 | No |
| 97 | AMACR | na |  |  | 15886 | -0.663 | -0.3987 | No |
| 98 | XBP1 | na |  |  | 16003 | -0.687 | -0.4003 | No |
| 99 | P2RX4 | na |  |  | 16172 | -0.726 | -0.4044 | No |
| 100 | CD81 | na |  |  | 16248 | -0.744 | -0.4035 | No |
| 101 | SOCS1 | na |  |  | 16397 | -0.781 | -0.4062 | Yes |
| 102 | RHOB | na |  |  | 16399 | -0.781 | -0.4012 | Yes |
| 103 | ADAM19 | na |  |  | 16535 | -0.820 | -0.4030 | Yes |
| 104 | EMP1 | na |  |  | 16599 | -0.834 | -0.4008 | Yes |
| 105 | GPX4 | na |  |  | 16614 | -0.837 | -0.3961 | Yes |
| 106 | MAFF | na |  |  | 16697 | -0.856 | -0.3949 | Yes |
| 107 | MYO1E | na |  |  | 16746 | -0.868 | -0.3917 | Yes |
| 108 | ETV4 | na |  |  | 16819 | -0.894 | -0.3897 | Yes |
| 109 | MAP3K8 | na |  |  | 16847 | -0.904 | -0.3852 | Yes |
| 110 | PLIN2 | na |  |  | 16877 | -0.913 | -0.3808 | Yes |
| 111 | ECM1 | na |  |  | 16989 | -0.949 | -0.3805 | Yes |
| 112 | IKZF4 | na |  |  | 16995 | -0.950 | -0.3746 | Yes |
| 113 | PIM1 | na |  |  | 17218 | -1.013 | -0.3796 | Yes |
| 114 | IGF1R | na |  |  | 17220 | -1.013 | -0.3731 | Yes |
| 115 | TNFRSF21 | na |  |  | 17238 | -1.017 | -0.3674 | Yes |
| 116 | IFNGR1 | na |  |  | 17299 | -1.042 | -0.3637 | Yes |
| 117 | SLC29A2 | na |  |  | 17352 | -1.060 | -0.3596 | Yes |
| 118 | CCND2 | na |  |  | 17385 | -1.070 | -0.3543 | Yes |
| 119 | IFITM3 | na |  |  | 17388 | -1.072 | -0.3474 | Yes |
| 120 | SPRY4 | na |  |  | 17489 | -1.119 | -0.3454 | Yes |
| 121 | CSF2 | na |  |  | 17557 | -1.135 | -0.3415 | Yes |
| 122 | CDKN1C | na |  |  | 17571 | -1.139 | -0.3348 | Yes |
| 123 | ITGAV | na |  |  | 17601 | -1.149 | -0.3289 | Yes |
| 124 | TLR7 | na |  |  | 17739 | -1.198 | -0.3283 | Yes |
| 125 | PRAF2 | na |  |  | 17840 | -1.249 | -0.3254 | Yes |
| 126 | CTSZ | na |  |  | 17841 | -1.251 | -0.3172 | Yes |
| 127 | P4HA1 | na |  |  | 18001 | -1.330 | -0.3169 | Yes |
| 128 | COL6A1 | na |  |  | 18070 | -1.364 | -0.3116 | Yes |
| 129 | IL4R | na |  |  | 18107 | -1.380 | -0.3045 | Yes |
| 130 | CDCP1 | na |  |  | 18121 | -1.385 | -0.2962 | Yes |
| 131 | CISH | na |  |  | 18183 | -1.416 | -0.2902 | Yes |
| 132 | ANXA4 | na |  |  | 18218 | -1.434 | -0.2827 | Yes |
| 133 | NFIL3 | na |  |  | 18225 | -1.442 | -0.2736 | Yes |
| 134 | WLS | na |  |  | 18263 | -1.464 | -0.2660 | Yes |
| 135 | NRP1 | na |  |  | 18351 | -1.527 | -0.2606 | Yes |
| 136 | SNX9 | na |  |  | 18365 | -1.539 | -0.2513 | Yes |
| 137 | BHLHE40 | na |  |  | 18387 | -1.556 | -0.2423 | Yes |
| 138 | SERPINB6 | na |  |  | 18462 | -1.616 | -0.2357 | Yes |
| 139 | TRAF1 | na |  |  | 18477 | -1.625 | -0.2258 | Yes |
| 140 | TNFSF10 | na |  |  | 18488 | -1.633 | -0.2157 | Yes |
| 141 | TIAM1 | na |  |  | 18560 | -1.701 | -0.2084 | Yes |
| 142 | ENPP1 | na |  |  | 18585 | -1.727 | -0.1984 | Yes |
| 143 | LTB | na |  |  | 18739 | -1.948 | -0.1938 | Yes |
| 144 | AHR | na |  |  | 18744 | -1.954 | -0.1813 | Yes |
| 145 | GBP4 | na |  |  | 18924 | -2.365 | -0.1753 | Yes |
| 146 | DHRS3 | na |  |  | 18963 | -2.502 | -0.1610 | Yes |
| 147 | NFKBIZ | na |  |  | 18992 | -2.610 | -0.1455 | Yes |
| 148 | SMPDL3A | na |  |  | 19011 | -2.710 | -0.1288 | Yes |
| 149 | SLC2A3 | na |  |  | 19034 | -2.803 | -0.1117 | Yes |
| 150 | TNFRSF9 | na |  |  | 19048 | -2.893 | -0.0936 | Yes |
| 151 | GABARAPL1 | na |  |  | 19059 | -2.914 | -0.0752 | Yes |
| 152 | CST7 | na |  |  | 19080 | -2.998 | -0.0567 | Yes |
| 153 | NDRG1 | na |  |  | 19083 | -3.028 | -0.0371 | Yes |
| 154 | RORA | na |  |  | 19112 | -3.279 | -0.0173 | Yes |
| 155 | BMP2 | na |  |  | 19119 | -3.339 | 0.0041 | Yes |
Table: GSEA details [plain text format]

  

Fig 2: HALLMARK\_IL2\_STAT5\_SIGNALING      
 Blue-Pink O' Gram in the Space of the Analyzed GeneSet

  

Fig 3: HALLMARK\_IL2\_STAT5\_SIGNALING: Random ES distribution      
 Gene set null distribution of ES for **HALLMARK\_IL2\_STAT5\_SIGNALING**
